# Age at surgery is correlated with pain scores following trochlear osteotomy in lateral patellar instability: a cross-sectional study of 113 cases

**DOI:** 10.1186/s13018-021-02485-4

**Published:** 2021-05-25

**Authors:** Jordy D. P. van Sambeeck, Nico Verdonschot, Albert Van Kampen, Sebastiaan A. W. van de Groes

**Affiliations:** Department of Orthopaedic Surgery, Radboudumc, PO Box 9101, 6500 HB Nijmegen, the Netherlands

**Keywords:** Patella, Patellofemoral joint, Patellar instability, Trochlear dysplasia, Trochlear osteotomy

## Abstract

**Background:**

A trochlear osteotomy aims to restore patellar stability in patients with recurrent patellar instability and trochlear dysplasia. The age of patients at time of surgery could be a relevant factor which influences outcome. We hypothesized that lower age at time of surgery is associated with better patient-reported outcomes.

**Methods:**

A retrospective study was conducted on patients with patellar instability and trochlear dysplasia. Patients were contacted by phone for informed consent and were then asked to complete online patient-reported outcome measurements (PROMs). The PROMs consisted of the Kujala Knee Score (KKS) (Kujala et al., Arthroscopy 9(2):159-63, 1993; Kievit et al. Knee Surg Sports Traumatol Arthrosc. 21(11):2647-53, 2013), the Short Form 36-item health survey (SF-36v1) (Ware, Med Care 73-83, 1992; Aaronson et al., J Clin Epidemiol. 51(11):1055-68, 1998), and visual analog scale (VAS) scoring pain, instability, disability, and satisfaction on a 0–100 scale. Multivariable linear regression models were used to study the effect of age on the PROM scores.

**Results:**

For this study, 125 surgical procedures in 113 patients were included. Mean VAS pain at rest was 19 and at activity 38; mean Kujala score was 73. Multivariable regression analysis revealed that age at the time of surgery was correlated with VAS pain at rest, with a 0.95 increase of VAS score (scale 0–100) for every year of age. Recurrence of instability was observed in 13 (10%) knees.

**Conclusion:**

In this cross-sectional study, pain scores of 113 patients who have undergone a lateral facet elevating trochlear osteotomy for patellar instability were reported. Age at time of surgery was correlated with an increased pain score at rest with an average of 9.5 points (scale 0–100) for every 10 years of age. Age at time of surgery was not correlated with overall satisfaction.

## Background

Patellar instability is a common problem seen by orthopedic surgeons. The annual incidence of primary patellar dislocation has been estimated at 43 per 100,000 in children under 16 years [1]. Recurrent patellar dislocation occurs in 15 to 45% of primary dislocation cases [2–7]. Patellofemoral stability is maintained by static stabilization of bony and soft tissue structures on the one hand and by dynamic stabilization through the activation of muscles on the other hand. The lateral displacement is statically restrained by the lateral facet of the trochlea and the medial patellofemoral ligament (MPFL) and mechanical alignment. Dynamic stabilization occurs by the activation and relaxation of co-acting muscles and muscle groups that directly or indirectly influence the position of the patella relative to the trochlea. Trochlear dysplasia is a condition in which the development of the trochlea results in an abnormal geometry, with a shallow, flat, or a convex shape of the sulcus. It has been identified as the most consistent anatomic factor present in patients with recurrent patellar dislocations [8]. In patients without pathoanatomical risk factors such as trochlear dysplasia, isolated MPFL reconstruction has a reliable outcome [9, 10]. A trochlear osteotomy could be added to the procedure for the surgical treatment of patients with recurrent patellar dislocations and trochlear dysplasia. Various surgical procedures have been described to reshape the abnormal trochlea [11, 12]. Trochlear osteotomies directly modify the patellofemoral joint with the risk of causing cartilage damage and alteration of joint kinematics and contact pressures [12]. Normal joint kinematics and contact pressures are fundamental for the long-term joint preservation, and abnormalities in these factors could potentially lead to development of early patellofemoral osteoarthritis [11–14]. Due to the presumed susceptibility of complications of a trochlear osteotomy, these procedures are not performed often. However, a systematic review of literature demonstrated that the rate of major complications is comparable to those of other patellar stabilizing procedures [15].

The indication for trochlear osteotomies is still a matter of debate. Combination of the procedure with another bony or soft tissue procedure is often necessary to achieve patellar stability throughout the full range of knee motion. Patellar stability is reported to be restored in a large majority of patients who had a trochlear osteotomy [11, 16, 17].

Next to the anatomical abnormalities such as trochlear dysplasia, patella alta, and insufficiency of the MPFL and MPTL [18], other patient factors such as their age could be of influence on the results of surgery for patellar instability. Multiple studies have shown that a correlation exists between the age of patients and the risk on recurrent dislocation and the outcomes of surgery. A study of Fithian et al. indicated that patients with initial injury at a younger age had a higher risk of subsequent patellar subluxation or dislocation [7]. Hiemstra et al. found a correlation between age at time of surgery (MPFL reconstruction) and outcome [19]. Palmu et al. [20] reported a redislocation rate of 67% following operative treatment for acute patellar dislocation in children younger than 16 years of age, which is much higher than the pooled risk of redislocation of 12% presented by Smith et al. [21] in their systematic review. Age at time of surgery might therefore be a factor for the risk of redislocation and patient-reported outcome of surgery. In addition to these correlations, adolescent patients are expected to have more pliable osseocartilaginous structures than the older patients [22]; this might lead to less cartilage damage by focal aseptic necrosis, better malleability, and therefore better clinical results in patients of a younger age.

The main goal of this study was to evaluate the effect of age on patient-reported outcome after a lateral facet elevating trochlear osteotomy in a large cohort.

We hypothesized that lower age at time of surgery is associated with better patient-reported outcomes.

## Methods

### Patients

Data for this study was collected retrospectively. Patients who have undergone a lateral facet elevating trochlear osteotomy to restore patellar stability in Radboudumc, Nijmegen, between 2005 and 2015 were included in this study. All operations were carried out by one senior orthopedic surgeon (AK) using the same surgical technique over time (described below). Indications for trochlear osteotomy were recurrent patellar dislocation or subluxation in the presence of a positive J-sign and radiographically confirmed trochlear dysplasia on a true lateral X-ray according to the criteria of Dejour and Saggin [23]. Radiologically closed epiphyses stage 3 or 4 (scale range 0–4) was confirmed in all patients; this was scaled according to the method described by O’Connor et al. [24]. Stage 3 indicates recent union, and stage 4 indicates complete union when remodeling has taken place and there is continuity of trabeculae form shaft to former epiphysis.

This study was approved by the Medical Ethical Review Board of the Radboudumc, Nijmegen, The Netherlands (CMO 2015-1943).

### Methods of assessment

The research team contacted each participant by phone to explain the study and have the participants complete patient-reported outcome measurements (PROMs). A secured website was used to complete reports. The PROMs included the Kujala Knee Score (KKS) [25, 26], the Short Form 36-item health survey (SF-36v1) [27, 28], and visual analog scale (VAS) scoring pain, instability, disability, and satisfaction. At time of surgery, no preoperative or postoperative PROMs were collected. Due to the retrospective nature of this study, those data are lacking and are not included in the analysis.

### Statistical analysis

Descriptive statistics was used to summarize the data. Multivariable linear regression models were used to evaluate the association between age (independent variable) and VAS and KKS scores (dependent variables). Based on clinical experience and published literature, we selected gender, history of surgery, presence of low or high grade trochlear dysplasia (low vs. high grade: A or C vs. B or D), postoperative patellar height, BMI, and whether or not additional procedures were performed as independent variables that could influence patient-reported outcome of surgery [29–31]. The multivariable regression analysis adjusts for these factors. Due to a lack of data, BMI as independent variable was not taken into account for analysis. A P-value of <0.05 was considered statistically significant. All statistical analyses were performed using SPSS (v20, IBM SPSS Statistics, Armonk, NY, USA).

### Surgical technique

**Fig. 1:**
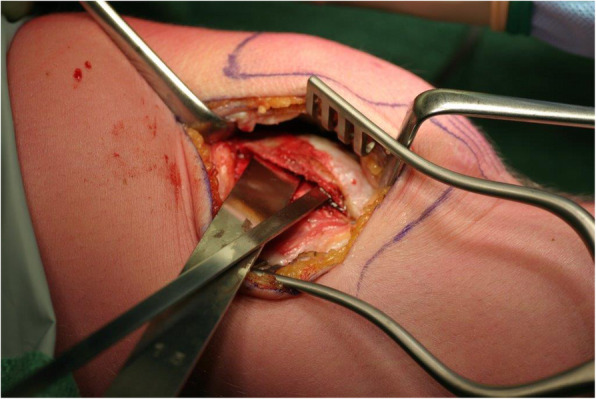
The osteotomy is performed with osteotomes; the proximal is further advanced medially than the distal osteotome

**Fig. 2:**
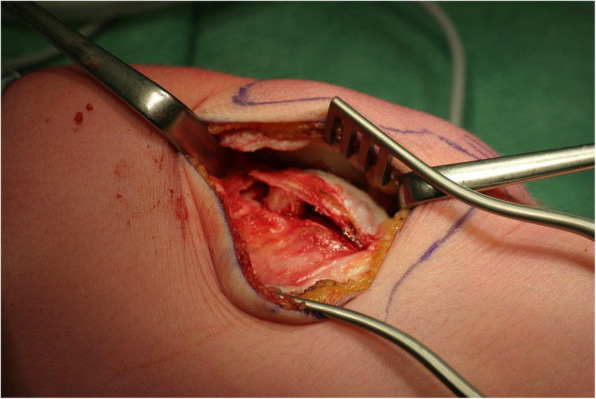
A triangular bone graft or tricalcium phosphate wedge is used to hold the achieved correction

A surgical technique was used as previously described by Koëter et al. [32] and slightly differs from the lateral facet elevating trochlear osteotomy as described by Albee and Weiker [33, 34]. In brief, the patient was placed supine on the table. Antibiotics were admitted preoperatively. No tourniquet was used. A lateral parapatellar incision was made and extended distally along the lateral femoral condyle. The retinaculum was opened in the direction of the femur. To visualize the osteotomy, two Kirschner wires were placed in the direction of the osteotomy till they were visible through the cartilage (halfway between the medial and lateral femoral facet). With the use of a small osteotome, an incomplete lateral trochlear osteotomy was carried out (Fig. 1). The curved osteotomy extended from the beginning of the trochlea proximally to the sulcus terminalis distally. Subsequently, the lateral articular surface of the trochlea was levered. In most cases, it was possible to raise the lateral articular surface by 4–6 mm. A wedge-shaped autograft was created with a part of the ipsilateral iliac crest to secure the elevation of the osteotomy; this graft was changed to a tricalcium phosphate (TCP) wedge during the study period (Fig. 2). Fixation of the osteotomy with osteosynthesis material was not needed. After performance of the osteotomy, the synovium was closed over while the lateral retinaculum was left open. Postoperatively, patients were placed on a continuous passive motion device (CPM) to stimulate a full passive range of motion until knee flexion was at least 60°. Patients were recommended the following training schedule: partial weight bearing for the first 6 weeks, without flexion limitation. After 6 weeks, full weight bearing was allowed. Patients were only referred to a physical therapist if restoration of normal gait was needed.

## Results

The patient database of our hospital identified 180 trochlear osteotomies in 150 unique patients, of whom 37 patients (55 procedures) could not be contacted or refused to participate. This led to the inclusion of 113 patients with 125 surgical procedures. Twelve patients had undergone bilateral trochlear osteotomy. Demographics and mean outcome scores are displayed in Table 1. Nine patients had no true lateral X-ray available in the database and were therefore not retrospectively classified into one of the four types of trochlear dysplasia.

**Table 1:** Demographic characteristics and outcome scores

Age at surgery in years (range)	19.8 (12.5–46.3)
Follow-up time (months)	71 (12–125)
Gender	
Female (%)	102 (82)
Type of trochlear dysplasia (%)	
Type A	14 (12)
Type B	55 (49)
Type C	9 (8)
Type D	33 (29)
Previous procedures (%)	16 (13)
Additional procedures (%)	69 (55)
MPFL reconstruction	7 (5.6)
Tibial tuberosity transfer	63 (50)
Complications during follow-up (%)	9 (7)
Flexion deficit	4 (3)
Persisting instability	1 (1)
Removal TCP wedge	2 (2)
Break-out of osteotomy	1 (1)
Venous thromboembolic event	1 (1)
Outcome score	
VAS pain at rest	19 (0–80)
VAS pain at activity	38 (0–90)
VAS instability	40 (0–100)
VAS disability	34 (0–100)
VAS satisfaction	66 (1–100)
Kujala Knee Score	73 (17–100)
SF-36 General health	72 (10–100)

Values represent mean with range, unless otherwise indicated. N is number of knees unless otherwise indicated

The mean VAS for pain at rest was 19 while this was 38 during activity. The mean VAS for overall satisfaction was 66. The mean KKS was 73, and the mean SF-36 general health perception was 72 (Table 1). In our cohort of patients with a mean age of 19.8 years (range 12.5–46.3) at surgery, multivariable regression analysis (Tables 2 and 3 and Tables 4, 5, 6 and 7 in the Appendix) revealed a correlation between age at time of surgery and VAS pain at rest. The VAS pain score at rest increased with 0.95 (0–100 scale) with every year of age at time of surgery (P 0.025). Recurrence of instability was seen in 13 (10%) knees.

**Table 2:** Results of multivariable regression analysis for VAS pain rest as dependent variable

Risk factor (independent variable)	Multivariable regression coefficient (95% CI)	***P***-value
**Age at time of surgery**	**0.95 (0.12, 1.8)**	**0.025**
**Female**	**12.7 (1.1, 24.3)**	**0.032**
Previous procedures performed	10.3 (−3.9, 24.5)	0.15
Patellar height	0.12 (−23.0, 23.2)	0.99
Trochlear dysplasia A or C vs. B or D	−2.5 (−13.8, 8.8)	0.66
Additional MPFL reconstruction	−2.8 (−24.7, 19.1)	0.80
Additional tibial tubercle transfer	−0.52 (−10.0, 9.0)	0.91

*CI* confidence interval

Bold risk factor: statistically significant

**Table 3:** Results of multivariable regression analysis for Kujala Knee Score as dependent variable

Risk factor (independent variable)	Multivariable regression coefficient (95% CI)	***P***-value
Age at time of surgery	−0.47 (−1.0, 0.17)	0.15
**Female**	−**11.6 (**−**20.4,** −**2.7)**	**0.011**
**Previous procedures performed**	**–16.2 (**−**27.1,** −**5.3)**	**0.004**
Patellar height	−11.6 (−29.2, 6.1)	0.20
Trochlear dysplasia A or C vs. B or D	2.6 (−6.1, 11.3)	0.55
Additional MPFL reconstruction	5.8 (−11.0, 22.6)	0.49
Additional tibial tubercle transfer	0.94 (−6.3, 8.2)	0.80

*CI* confidence interval

Bold risk factor: statistically significant

Complications included a postoperative flexion deficit in four knees. In one knee of a female patient at the age of 47 years at time of surgery, postoperatively, the trochlear osteotomy broke out to the distal femoral condyle; open reduction and refixation with two screws was performed; however, it resulted in arthrofibrosis and patellofemoral osteoarthritis (PF OA). One patient had persisting instability together with PF OA in the knee and underwent a patellofemoral arthroplasty 1 year postoperatively; in two patients, the tricalcium phosphate (TCP) wedge was removed because of dislocation, in one patient it dislocated, and in one patient this wedge broke, and one patient had deep venous thrombosis.

## Discussion

In this study, we showed that a VAS pain score increased with higher age after a lateral facet elevating trochlear osteotomy. Although the increase is relatively small for every year of age (0.95), it is a clinically relevant increase for every 10 years of age (9.5).

This study was not designed to investigate the underlying cause of increased pain; however, the following hypothesis could well be true. Older patients have a longer history of patellofemoral instability (peak incidence of first dislocation is in adolescence [7]). They might have a history of patellar dislocations with a longer period of maltracking of the patella. As a consequence, increased cartilage damage and degenerative changes of the patellofemoral joint would be present at time of surgery. This might have an influence on the level of correction that is possible, the congruency of the new trochlea, the presence of microtears during surgery, and the load-bearing capacity of the cartilage. These factors could also be influenced by the pliability of the articular cartilage, which decreases with increasing age due to molecular changes. Despite the fact that increasing age is correlated with a higher VAS pain score at rest in our study, we do not assume that this is caused by early PF OA. Although the present study did not evaluate the radiological presence of PF OA, a study by Tigchelaar et al. [35] showed no clear correlation between VAS pain and the grade of PF OA after trochlear osteotomy. They analyzed data from patients with 12 years of follow-up. Radiological PF OA after surgery was generally limited to lower grades on the Iwano scale [35]. In our study, two older patients (age at surgery 27 and 46 years) had radiologically confirmed PF OA during follow-up; this was radiologically examined because of the presence of persisting pain. Both patients scored high on VAS pain at rest and therefore contributed to an overall increased average in our study. Early symptomatic PF AO is not expected to be the main reason for an increase in mean VAS score at higher age. We hypothesize that decreased adaptation of the cartilage to the new situation results in increased subchondral pressure and higher pain scores at rest.

Age at time of surgery did not have a significant effect on other outcome measurements. However, it should be noticed that a relatively high rate of redislocation after trochlear osteotomy (10%) was found. Age of these patients ranged between 12 and 22 years with an average of 16 years and is lower than the average age of patients in our cohort (19.8 years). Furthermore, results of the multivariate linear regression analysis revealed significantly higher VAS instability score in females vs. males. With the exception of gender, no other risk factors were significantly correlated with VAS instability. In our cohort, an additional MPFL reconstruction was only performed in 5.6% of patients. Recently published literature and new surgical techniques for MPFL reconstruction have narrowed the indication for an isolated trochlear osteotomy without MPFL reconstruction. An additional MPFL reconstruction probably decreases the rate of redislocation. With this in mind, a redislocation rate of 10% can be seen as proof of the effectiveness of a trochlear osteotomy in terms of stability.

The mean KKS in our study was 73; this is an acceptable score in our opinion. The systematic review of Balcarek et al. demonstrates higher KKS scores after trochleoplasty procedures (range 81–92) [36]. However, due to the inclusion and exclusion criteria (for example the exclusion of studies in which treatment included additional procedures) of their review, it is difficult to compare our cohort with the studies they have included.

Loss in range of motion occurred in four patients (2%), which was lower in our study than in previously published reports [15]. The standard use of a CPM might have contributed to this. Four patients had a complication related to the TCP wedge.

The results of our study, including a relatively high rate of patellar redislocation and KKS of 73, emphasize that patient selection and strict indication for this type of trochleoplasty is highly important for a better outcome. A recent consensus statement from the AOSSM/PFF Patellofemoral Instability Workshop as well as recent guidelines stated that trochleoplasty is rarely indicated in patients with patellar instability [37, 38]. In determining whether surgery for recurrent patellar instability is warranted, trochlear morphology, patellar height, lateralization of the tibial tubercle, sufficiency of the MPFL, age, and gender should be considered. None of the different types of dysplasia according to Dejour and Saggin was correlated with outcome measurements of this study. The indications for a lateral facet elevating trochlear osteotomy have decreased in recent years. However, we think that there is still a place for this type of trochlear osteotomy in patients with recurrent patellar dislocation with a J-sign at physical exam, underlying trochlear dysplasia without a trochlear bump but with a convex proximal trochlea. In these cases, outcomes are most predictable, and the risk of serious complications is low.

This is the first study to investigate the effect of age on the postoperative outcomes of a lateral facet elevating trochlear osteotomy. The strength of this study is the large patient cohort and the use of PROMs which reflect the outcome as experienced by patients and not based on radiographs assessed by clinicians. Despite the minimal number of indications, we are convinced that it is important to present the outcomes of this type of trochlear osteotomy, studied in a large cohort, in the perspective of personalized treatment for the individual patient.

Our study also has some potential limitations. First, our study population was heterogenic (e.g., different types of dysplasia, multiple additional procedures). Although intrinsic heterogeneity in patient characteristics and treatment strategy exists in this study, we think that this population reflects the patients seen during daily practice. Second, patients were not physically examined by a clinician. Our study demonstrates that patient-reported outcomes, in conjunction with surgical complications, are most relevant to determine post-surgical outcome. This warrants the use of PROMs in this patient category. Third, a difference in the level of preoperative chondropathy between younger and older patients could influence postoperative outcome, but a quantifiable report on the preoperative chondral status is lacking in our study. A lot of patients with patellar luxation or subluxation have some amount of patellofemoral chondropathy; this is inherent to the underlying pathology. It has not been demonstrated that preoperative chondral status is associated with postoperative outcome. However, a surgeon should be aware of the possible interaction of chondral status and outcome of surgery.

## Conclusion

In this cross-sectional study, pain scores of 113 patients who have undergone a lateral facet elevating trochlear osteotomy for patellar instability were reported. Age at time of surgery was correlated with an increased pain score at rest with an average of 9.5 points (scale 0–100) for every 10 years of age. Age at time of surgery was not correlated with overall satisfaction.

## Data Availability

The datasets used and/or analyzed during the current study are available from the corresponding author on reasonable request.

## Appendix

**Table 4:** Results of multivariable regression analysis for VAS pain activity as dependent variable

Risk factor (independent variable)	Multivariable regression coefficient (95% CI)	***P***-value
Age at time of surgery	0.79 (−0.23, 1.8)	0.13
**Female**	**21.0 (6.8, 23.3)**	**0.004**
Previous procedures performed	14.8 (−2.7, 32.2)	0.096
Patellar height	9.9 (−18.5, 38.3)	0.49
Trochlear dysplasia A or C vs. B or D	−8.0 (−21.9, 6.0)	0.26
Additional MPFL reconstruction	1.8 (−25.1, 28.8)	0.89
Additional tibial tubercle transfer	−4.4 (−16.0, 7.4)	0.46

*CI* confidence interval

Bold risk factor: statistically significant

**Table 5:** Results of multivariable regression analysis for VAS instability as dependent variable

Risk factor (independent variable)	Multivariable regression coefficient (95% CI)	***P***-value
Age at time of surgery	−0.22 (−1.4, 0.96)	0.71
**Female**	**17.2 (0.58, 33.8)**	**0.043**
Previous procedures performed	7.6 (−12.7, 27.8)	0.46
Patellar height	19.4 (−13.6, 52.4)	0.25
Trochlear dysplasia A or C vs. B or D	4.9 (−11.3, 21.2)	0.55
Additional MPFL reconstruction	−1.5 (−32.9, 29.8)	0.92
Additional tibial tubercle transfer	1.4 (−12.2, 15.0)	0.84

*CI* confidence interval

Bold risk factor: statistically significant

**Table 6:** Results of multivariable regression analysis for VAS disability as dependent variable

Risk factor (independent variable)	Multivariable regression coefficient (95% CI)	***P***-value
Age at time of surgery	0.13 (−1.0, 1.3)	0.82
Female	8.6 (−7.4, 24.6)	0.29
Previous procedures performed	17.1 (−2.5, 36.7)	0.086
Patellar height	10.6 (−21.2, 42.4)	0.51
Trochlear dysplasia A or C vs. B or D	10.5 (−5.2, 26.1)	0.19
Additional MPFL reconstruction	−12.7 (−42.9, 17.5)	0.41
Additional tibial tubercle transfer	2.1 (−11.1, 15.2)	0.76

*CI* confidence interval

Bold risk factor: statistically significant

**Table 7:** Results of multivariable regression analysis for VAS satisfaction as dependent variable

Risk factor (independent variable)	Multivariable regression coefficient (95% CI)	***P***-value
Age at time of surgery	−0.25 (−1.5, 1.0)	0.70
**Female**	−**22.6 (**−**40.2,** −**4.9)**	**0.013**
Previous procedures performed	−11.6 (−33.2, 10.0)	0.29
Patellar height	−7.5 (−42.7, 27.6)	0.67
Trochlear dysplasia A or C vs. B or D	2.8 (−14.5, 20.1)	0.75
Additional MPFL reconstruction	5.3 (−28.0, 38.7)	0.75
Additional tibial tubercle transfer	−2.2 (−16.7, 12.3)	0.76

*CI* confidence interval

Bold risk factor: statistically significant

## Abbreviations

CI: Confidence interval
CPM: Continuous passive motion
IRB: Institutional Review Board
KKS: Kujala Knee Score
MPFL: Medial patellofemoral ligament
NRS: Numeric rating scale
PF: Patellofemoral
PF OA: Patellofemoral osteoarthritis
PROMs: Patient-reported outcome measurements
SF-36: Short form 36-item Health Survey
TCP: Tricalcium phosphate
VAS: Visual analog scale
